# Effects of G.H.3. On mental symptoms and health-related quality of life among older adults: results of a three-month follow-Up study in Shanghai, China

**DOI:** 10.1186/s12937-016-0133-5

**Published:** 2016-01-26

**Authors:** Gang Xu, Zhaochun Cao, Mina Shariff, Pingping Gu, Tuong Nguyen, Tian Zhou, Rong Shi, Jianyu Rao

**Affiliations:** 1 School of Public Health, Shanghai Jiaotong University, Shanghai, China; 2 Jiaxing Community Health Service Center, Hongkou District Shanghai, China; 3 Department of Research, DRM Resources, 1683, Sunflower Avenue, Costa Mesa, CA 92626 USA; 4 Beijing University of Chinese Medicine, Beijing, China; 5 Department of Pathology and Laboratory Medicine, in UCLA Medical Center, University of California, Los Angeles, CA USA

**Keywords:** G.H.3, Mental Symptoms, Health-Related Quality of Life, Older Adults, Low Mood, Anxiety, Double-blinded, SDS, SAS, SF-36

## Abstract

**Objective:**

To explore the effects of daily use of Gerovital H3 (G.H.3.) tablets on relieving mental symptoms and improving health-related quality of life among Chinese older adults population.

**Methods:**

In a randomized, placebo-controlled, double-blinded study, totally 100 eligible participants were randomly allocated into the G.H.3. group or the placebo group, administered either G.H.3. or placebo tablets and were followed up for three months. All of the participants were required to report their subjective feelings about quality of life, low mood, and anxiety by filling out Self-Rating Depression Scale (SDS), Self-Rating Anxiety Scale (SAS) and a 36-item Short-Form Health Survey (SF-36 scale). Physicians were responsible for evaluating the related mental health indications through physical examinations at the baseline and at the end of the intervention period.

**Results:**

Participants were men and women between 50 and 89 years of age, with a median of 62.53 years. Before the intervention, the demographic characteristics and the baseline SF-36 scores, low mood, and anxiety statuses were comparable (*p* > 0.05). After the 12-week intervention, the scores of role-physical (RP), bodily pain (BP), general health (GH), vitality (VT), mental health (MH) and health transition (HT), mental composite score (MCS) of the G.H.3. group were higher than the placebo group (*p* < 0.05), There were no significant differences in other domains in SF-36 and PCS between the two groups(*p* > 0.05), the scores of SDS and SAS in the G.H.3. group were both lower than the placebo group(*p* < 0.01), the prevalence rates of low moods in the G.H.3. group and the placebo group were 20.8 % and 34.0 % respectively, no significant difference was found (*χ* ^2^ =2.127,*p* = 0.145), while the prevalence rate of clinical anxiety concerns in the G.H.3. group was 2.1 %, which was significantly lower than the placebo group, 22.0 % (*χ* ^2^ =9.040,*p* < 0.001).

**Conclusions:**

Preliminarily use of G.H.3. shows positive effects in supporting mental health and improving general health and well-being while promoting the recovery of cognitive function among older adults. Most of SF-36 domains including PF, RP, BP, GH, VT, RE, MH, and HT, as well as the overall quality of life in MCS might benefit from taking G.H.3. tablets. Average levels of low moods and anxiety concerns were both reduced and the prevalence rate of clinical anxiety concerns were reduced.

## Introduction

The quality of life in older adults is largely determined by their functional status and health conditions [1]. Since 1999, China has entered the aging society, by 2010, China's elderly population over 60 years of age has reached to 0.178 billion, accounting for 13.3 % of the whole country's population, the figure of Chinese elderly population will still increase steadily by 8 million per year in the near future [2].

When adults enter middle-age, a reduction of intracranial neurotransmitters, cerebral blood flow, cerebral oxygen consumption, and catecholamine can make them more prone to memory and cognitive dysfunction [3], poor sleep, low moods, anxiety, frequent insomnia, fatigue, dizziness, absent-mindedness, response slowly, headaches, tinnitus, and other signs associated with aging [4–8]. These factors can affect the quality of life in elderly people to a large extent, and may even indirectly lead to other chronic diseases, such as hypertension [9] and coronary heart disease [10].

Gerovital H3 (G.H.3.) (Manufactured by Robinson Pharma, Inc., Orange County, CA, USA) is a nutritional supplement that may provide potential effects in improving mental health and improving health-related quality of life. The current experiment performed was a randomized, double-blind, placebo-controlled study conducted on older adults in China experiencing at least one of the following symptoms/complaints which included anxiety, inattention, memory decline, insomnia, self-induced fatigue, sleepiness, slow responses, involuntary staring, absent-mindedness, vertigo, dizziness, headaches, tinnitus, palpitations, chest pain, etc.

As the first G.H.3. randomized controlled trial(RCT) research conducted on Chinese older adults populations, the objective of this study was to explore the effects of daily use of G.H.3. tablets in improving  mental health and improving health-related quality of life.

## Methods

### Subjects

A total of 122 participants were recruited from the outpatient department of Jiaxing Community Health Service Center, Hongkou District, Shanghai, China, from November 19, 2011, to December 21, 2011.

All of the participants were men and women over 50 years old who had at least one of the related complaints associated with mental health and health-related life quality decline which included anxiety, inattention, memory decline, insomnia, self-induced fatigue, sleepiness, response slowly, involuntary staring, absent-mindedness, vertigo, dizziness, headaches, tinnitus, palpitations, chest pain, etc. The study excluded 16 individuals who had Alzheimer’s Disease, severe depression, mental illness, cancer of any type, history of any severe disease diagnosis (including severe diabetes, cardiovascular disease, etc.) or those currently (within 30 days) taking any prescription or over-the-counter drugs that may affect neuroendocrine function. We also excluded 6 individuals who were eligible but had refused to join the study, so there were totally 100 participants took part in this randomized, placebo-controlled, double-blinded study at the beginning.

Using random number tables, the 100 participants who met all the eligibility criteria provided written, informed consent prior to the study and were randomly allocated into the G.H.3. group (Experimental, *n* = 50) or the placebo group (Control, *n* = 50) with simple randomization method. Participants were administered either G.H.3. or placebo tablets and were followed up for three months. The determination of sample size was mainly based on the limitations of research period and research funds.

### Intervention

Each participant in both groups received a container marked with different-colored labels (which was blinded for both subjects and researchers) and detailed instructions for administrating the tablets. The unmasking was done at the end of the intervention.

The G.H.3. and the placebo tablets were both provided by Robinson Pharma, Inc (Orange County, CA, USA). Participants in the experimental group (G.H.3. group) and in the control group (placebo group) both took two placebo tablets orally, once daily. The active components of the G.H.3. tablets were comprised of Vitamin A, vitamin C, vitamin E, vitamin B1, vitamin B2, vitamin B3, vitamin B6, folic acid, vitamin B12, calcium, magnesium, zinc, selenium, chromium, St. John’s wort (*Hypericum perforatum*), Dimethylaminoethanol (DMAE), para-aminobenzoic acid (PABA), *Ginkgo biloba*, and L-glutathione. The main ingredient of the placebo tablets was flour.

**Fig. 1:**
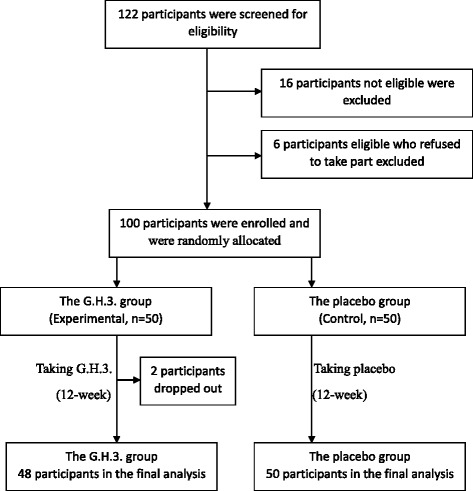
Flow diagram of the study design

The total intervention period lasted for 12 weeks. Two subjects in the G.H.3. group dropped out in the first month, because their relatives refused to let them continue taking the tablets, resulting in 98 participants remaining who were followed up once per month. Tablets were dispensed with follow-ups. All follow-ups for both the G.H.3. group and the placebo group were completed on March 14, 2012. A total of 98 cases completed the last follow-up and final survey, (48 cases in the G.H.3. group, 50 cases in the placebo group) (see Fig. 1).

### Assessment of outcomes

All patients were required to complete a questionnaire which recorded their date of birth, gender, ethnicity, medical history, smoking history, alcohol intake history, medical history, current medication(s), and complaints relating to mental health at the baseline. All of the participants were also required to report their subjective feelings about quality of life, low moods, and current anxiety levels (within past week) by filling out three measurement scales which included surveys for Self-Rating Depression Scale (SDS), Self-Rating Anxiety Scale (SAS) and a 36-item Short-Form Health Survey (SF-36 scale) at the beginning of enrollment and one week after they finished taking all of the tablets.

At the baseline and at the end of the intervention period, physicians evaluated the related mental health indicators (including anxiety, inattention, memory decline, insomnia, self-induced fatigue, sleepiness, involuntary staring, slow responses, absent-mindedness, excessive use of brains, forgetfulness, vertigo, dizziness, headaches, tinnitus) for each participant through physical examinations, assigning scores ranging from 1 to 5, with 1 indicating the absence of a given concern and 5 indicating a severe degree of that specific concern. In order to evaluate the magnitude of symptom improvements among the participants in the G.H.3. group, we figured out the score changes of the same person before and after intervention. If the score of a mental health indicator after three-month follow-up reduced by 1 point or more compared to the beginning of the intervention, it was defined as an improvement.

The SDS and SAS scales each contain 20 items reflecting different indications of low mood and anxiety [11] and use a 4-point scoring method, participants gave an answer according to whether the item has occurred and its frequency: 1 = never/very rarely/rarely; 2 = once in a while/some of the time/occasionally; 3 = relatively often/very often/often; 4 = most of the time/always/almost always [12]. The original total scores of low moods and anxiety were obtained by summing up all of the scores of these 20 items (rang 20–80), then we converted them into SDS scores and SAS scores by using original score × 1.25(rang 25–100) [13]. Higher SDS scores and SAS scores generally reflected more serious levels of low moods and anxiety concerns. [14] The cutoff point of SAS score 50 and SDS score 53 considered as the presence of clinical anxiety concern and low moods respectively in Chinese population [15]. Several domestic and international research results show that both SDS and SAS surveys have high reliability and validity [16–18].

The SF-36 scale is a widely recognized health-related, quality-of-life (HRQoL) assessment tool used in Europe and America to evaluate both physical and mental health [19]. SF-36 scale includes eight domains, including physical functioning (PF), role-physical (RP), bodily pain (BP), general health (GH), vitality (VT), social functioning (SF), role-emotional (RE), and mental health (MH). In addition, there is a single-item indicator—health transition (HT) to assess the health changes in the past year [20]. Items were summed per domain and transformed into scores between 0–100, with higher values representing better function [21]. Physical composite score (PCS) and mental composite score (MCS) were calculated according to each domain score and the data of Chinese adults model [22]. PCS was significantly associated with chronic disease, walking ability, visual ability, sleep quality, marital status, alcohol consumption, hearing ability, smoking, neighborhood relationships, filial piety, ethnicity, and dietary habits. MCS was associated with chronic disease, sleep quality, walking ability, visual ability, marital status, ethnicity, filial piety, dietary habits, alcohol consumption, smoking, and hearing ability [2].

Along with the follow-ups, self-reported side effects and adherences were recorded.

The primary outcomes included the scores of the eight domains in SF-36(PF, RP, BP, GH, VT, SF, RE, MH), the single-item indicator—HT and the side effects. The secondary outcomes included the scores of PCS, MCS, SDS, SAS, the prevalence rate of low moods, the prevalence rate of clinical anxiety concerns and the improvement rates on the scores of mental symptoms.

### Statistical analysis

EpiData 3.02 software was used to establish the database. SPSS 20.0 software was used for statistical analysis. Variables were compared between the two groups by applying Student’s *t*-test for quantitative variables and Chi-square test for categorized variables. The alpha level chosen was 0.05. All p-values reported were 2-sided.

## Results

### Baseline data

#### The baseline demographic characteristics

Participants were men and women between 50 and 89 years of age, with a median of 62.53 years. The baseline demographic characteristics between the two groups were comparable on age, gender, alcohol intake history, etc. (see Table 1).

**Table 1:** Demographic characteristics for the G.H.3. group and the placebo group at baseline

Demographic characteristics	Frequency (percent)		*Total*	*Statistics/p*-value
	G.H.3. (*n* = 48)	Placebo (*n* = 50)		
Gender				
Male	21(43.8)	19(38.0)	40(40.8)	*χ* ^2^ =0.335,*p* = 0.563
Female	27(56.2)	31(62.0)	58(50.2)	
Age group(in years)				
50-59	16(33.3)	18(36.0)	34(34.7)	*χ* ^2^ =0.139,*p* = 0.933
60-69	17(35.4)	16(32.0)	33(33.7)	
70-	15(31.3)	16(32.0)	31(31.6)	
Alcohol intake				
Yes	12(25.0)	8(16.0)	20(20.4)	*χ* ^2^ =1.221,*p* = 0.269
No	36(75.0)	42(84.0)	78(79.6)	

#### Comparisons on SF-36 scores at baseline

The PCS, MCS, and scores of each domain between the G.H.3. group and the placebo group were compared and there was no significant difference being found (see Table 2).

**Table 2:** Comparisons on SF-36, SDS and SAS scores at baseline

Domain	Average scores ($\overline{X}\pm S$)		*t*	*p*-value
	G.H.3. (*n* = 48)	Placebo (*n* = 50)		
PF	69.27 ± 26.62	73.10 ± 27.14	−0.705	0.483
RP	41.67 ± 42.32	46.50 ± 41.04	−0.574	0.567
BP	70.42 ± 14.14	72.20 ± 15.82	−0.588	0.558
GH	42.71 ± 19.16	43.20 ± 18.32	−0.130	0.897
VT	56.46 ± 22.14	57.30 ± 20.21	−0.197	0.845
SF	66.41 ± 24.48	69.00 ± 21.91	−0.553	0.581
RE	45.14 ± 39.79	51.33 ± 38.81	−0.780	0.437
MH	57.58 ± 21.26	58.32 ± 19.23	−0.180	0.857
HT	36.98 ± 23.06	35.50 ± 17.56	0.358	0.721
PCS	40.61 ± 13.52	42.11 ± 14.57	−0.525	0.600
MCS	35.02 ± 17.93	36.14 ± 17.02	−0.318	0.751
SDS	54.97 ± 12.27	53.03 ± 11.16	0.823	0.412
SAS	48.88 ± 11.86	46.00 ± 10.22	1.289	0.200

#### Comparisons on low moods and anxiety statuses at baseline

Before the intervention, there was no significant difference between the G.H.3. group and the placebo group in the scores of SDS and SAS (see Table 2).

The prevalence rates of low moods in the G.H.3. group and the placebo group were 56.3 % (27/48) and 52.0 % (26/50) respectively, with no significant difference(*χ* ^2^ =0.178,*p* = 0.673). And the prevalence rates of clinical anxiety concerns in the G.H.3. group and the placebo group were 41.7 % (20/48) and 40.0 % (20/50) respectively, with no significant difference either(*χ* ^2^ =0.028,*p* = 0.867).

### Results after intervention

#### Comparisons on SF-36 scores after the intervention

This study also showed that participants in the G.H.3. group experienced better quality of life after taking the G.H.3. tablets compared to those taking the placebo. After the 12-week intervention, the score of RP in the G.H.3. group was 77.60 ± 35.45, which was significantly higher than the score (60.00 ± 38.13) in the placebo group (*p* = 0.020). The score of BP in the G.H.3. group was 81.25 ± 11.23 and was also significantly higher than the score (75.40 ± 14.17) in the placebo group (*p* = 0.026). The score of GH in the G.H.3. group was 56.77 ± 18.49 and was also significantly higher than the score (46.20 ± 17.63) in the placebo group (*p* = 0.005). The score of VT in the G.H.3. group was 72.92 ± 12.88 and was also significantly higher than the score (62.10 ± 17.16) in the placebo group (*p* = 0.001). There were also significant differences in the scores of MH and HT between the two groups. The score of MCS in the G.H.3. group was higher than which in the placebo group (*p* = 0.011). There was insufficient evidence to distinguish the effects of G.H.3. from the placebo for other domains and PCS (see Table 3).

**Table 3:** Comparisons on SF-36, SDS and SAS scores after the intervention

Domain	Average scores ($\overline{X}\pm S$)		*t*	*p*-value
	G.H.3. (*n* = 48)	Placebo (*n* = 50)		
PF	75.73 ± 22.17	72.90 ± 24.68	0.596	0.552
RP	77.60 ± 35.45	60.00 ± 38.13	2.365	0.020
BP	81.25 ± 11.23	75.40 ± 14.17	2.259	0.026
GH	56.77 ± 18.49	46.20 ± 17.63	2.897	0.005
VT	72.92 ± 12.88	62.10 ± 17.16	3.544*	0.001
SF	75.78 ± 23.55	70.50 ± 22.69	1.131	0.261
RE	81.25 ± 31.44	67.33 ± 38.39	1.959	0.053
MH	71.33 ± 13.96	62.16 ± 14.88	3.144	0.002
HT	53.65 ± 23.06	44.00 ± 20.55	2.188	0.031
PCS	45.88 ± 12.62	42.92 ± 14.23	1.088	0.279
MCS	48.09 ± 9.89	41.23 ± 15.73	2.596*	0.011
SDS	42.79 ± 8.65	49.68 ± 10.52	−3.532	0.001
SAS	37.89 ± 7.08	43.68 ± 8.42	−3.675	0.000

*: *t’*-test

#### Comparisons on low moods and anxiety after the intervention

After the intervention, the scores of SDS and SAS in the G.H.3. group were both lower than the placebo group (see Table 3).

The prevalence rate of low moods in the G.H.3. group was 20.8 % (10/48), no significant difference was found in the prevalence rate of the placebo group, which was 34.0 % (17/50) (*χ* ^2^ =2.127,*p* = 0.145), while the prevalence rate of clinical anxiety concerns in the G.H.3. group was 2.1 %(1/48), which was significantly lower than which in the placebo group, 22.0 % (11/50), (*χ* ^2^ =9.040,*p* < 0.001).

### The changes before and after the intervention in the G.H.3. group

#### SF-36 scores

All of the eight-domain scores in SF-36 were significantly improved in the G.H.3. group as were the scores for PCS and MCS(see Table 4) after the intervention.

**Table 4:** The changes of SF-36, SDS and SAS scores before and after the intervention in the G.H.3. group

Domain	Before (*n* = 48)	After (*n* = 48)	Difference	*t*	*p*-value
PF	69.27 ± 26.62	75.73 ± 22.17	6.46 ± 18.76	2.385	0.021
RP	41.67 ± 42.32	77.60 ± 35.45	35.94 ± 44.03	5.654	0.000
BP	70.42 ± 14.14	81.25 ± 11.23	10.83 ± 13.18	5.694	0.000
GH	42.71 ± 19.16	56.77 ± 18.49	14.06 ± 17.28	5.638	0.000
VT	56.46 ± 22.14	72.92 ± 12.88	16.46 ± 22.64	5.036	0.000
SF	66.41 ± 24.48	75.78 ± 23.55	9.38 ± 25.46	2.551	0.014
RE	45.14 ± 39.79	81.25 ± 31.44	36.11 ± 38.80	6.449	0.000
MH	57.58 ± 21.26	71.33 ± 13.96	13.75 ± 23.18	4.110	0.000
HT	36.98 ± 23.06	53.65 ± 23.06	16.67 ± 22.08	5.229	0.000
PCS	40.61 ± 13.52	45.88 ± 12.62	5.27 ± 10.44	3.497	0.001
MCS	35.02 ± 17.93	48.09 ± 9.89	13.07 ± 16.43	5.515	0.000
SDS	54.97 ± 12.27	42.79 ± 8.65	−12.19 ± 15.11	−5.588	0.000
SAS	48.88 ± 11.86	37.89 ± 7.08	−10.99 ± 14.54	−5.237	0.000

*: Paired-samples *t*-test

#### Low mood and anxiety statuses

The scores of SDS and SAS in the G.H.3. group were both reduced significantly after the intervention (see Table 4).

#### The improvement rates on the scores of mental health indicators

We found that there were totally six indicators of which improvement rates exceeding 50 %, including insomnia(60.4 %), dizziness(60.4 %), memory decline(54.2 %), self-induced fatigue(54.2 %), forgetfulness(52.1 %), and inattention(50.0 %).

### Side effects

No serious, adverse reaction from taking G.H.3. was found in the study, only one participant(Male, 58 years old) in the G.H.3. group reported that he had felt a slight bloating and nausea occasionally after taking G.H.3., but it was not serious and the concerns were soon relieved. One other person (Female, 59 years old) in the placebo group reported that she had felt a mild headache.

## Discussion

China has the world’s largest elderly population, meanwhile, the proportion of the elderly population continues to increase, the burden of the elderly related diseases are becoming more and more serious at the same time, for exsample, the results of some meta analyses show that the prevalence of clinical anxiety symptom and clinical depression symptom among the elderly in China has reached to 22.1 % [23] and 22.6 % [24], respectively. According to sleep disorder, the prevalence has even reached to 47.2 % [25], while sleep disorder was reported as a major risk factor of the morning peak phenomenon for an elderly patient with simple systolic hypertension, so improving the sleep quality is extremely essential for older people [26]. Maintaining and improving the quality of life of the elderly has also emerged as a particularly important issue [27].

G.H.3. was found to have positive effects in improving parts of mental health indicators such as inattention, memory decline, insomnia, self-induced fatigue, response slowly, absent-mindedness, forgetfulness, dizziness, headaches, etc. The lessening of these indicators will bring great benefits for the general health and state of well-being among older adults population. Between all of the related mental health indicators, insomnia was the most obviously improved. As mentioned earlier, improving sleep quality and reducing the frequency of sleep disorder is potentially very useful for the control of systolic blood pressure level in the morning, and the reduction of the cardiovascular events occurrence among elderly. The improvements of the memory decline, forgetfulness and inattention will potentially promote the recovery of cognitive function for the older adults and reduce the care giving burden of their families and communities.

It was also observed that most SF-36 domains including PF, RP, BP, GH, VT, RE, MH, and HT might benefit from taking G.H.3. tablets. MCS was found not only a significant difference between the G.H.3. group and the placebo group after the intervention, but also a great increase in the same G.H.3. group before and after, it indicates that mental health status might actually get improved after exclusion of placebo effect. Generally, PCS does not exhibit a significant difference between the G.H.3. group and the placebo group after the intervention, so there is no evidence can prove G.H.3. tablets help to improve physical health status in general.

G.H.3. preliminarily exhibited potent effects in improving the general degrees of low moods and anxiety concerns among older adults. It might also help to reduce prevalence rate of clinical anxiety concerns, but we didn’t observed that G.H.3. tablets could help to reduce prevalence rate of low moods. Due to these benefits mentioned above we may expect that the incidence risk of some chronic diseases, such as hypertension and coronary heart disease will be lowered indirectly to some extent.

Taking two tablets of G.H.3. orally once daily is considered to be safe due to no serious, adverse reaction being observed during the whole follow-up period in this study.

## Limitation

There were several limitations in this study. Firstly, because the outcome measured in this research was mainly based on subjective symptoms, the judgment of intervention effects could be easily influenced by psychological effects of the subjects and the researchers. Improvements of indicators in the placebo group after the intervention showed this effect, but the strict using of randomization, placebo-control, and double-blinded methods ensured a relatively higher validity of the results in this study.

Secondly, some potential confounding factors—such as weight, lifestyle, and dietary habits—were not controlled in the study. But as the two groups were well-balanced in the baseline characteristics, we expected that the effects of differences in these, or other potential confounding factors, are minimal.

Thirdly, the information from SF-36, SDS, SAS were obtained based on the participants’ recall, recall errors might exist, however, since the two groups had good comparability due to the double-blinded design, the possibility of recall bias should be relatively low.

Fourthly, all of our participants were volunteers, i.e. those eligible who did not wish to participate were excluded. If those who were excluded were not exactly compatible with those enrolled for the study, the results would suffer a selection bias.

Finally, because of the limitation of the study period and the sample size, which ensured a high hypothesis testing level(0.05), the efficiency of some hypothesis tests may not be very high.

The research has achieved the expected research purposes, G.H.3. preliminarily shows positive effects in improving parts of mental health, the average levels of low moods and anxiety concerns in participants, as well as improving general health-related quality of life factors, particularly in mental health levels among older adults. Further study is needed to confirm these inferences to a greater extent.

## Conclusions

In summary, G.H.3. preliminarily shows positive effects in improving parts of mental health such as inattention, memory decline, insomnia, self-induced fatigue, slow response, absent-mindedness, forgetfulness, dizziness, headaches, etc. and improving the general health and state of well-being, which promotes the cognitive function among older adults. Most of SF-36 domains including PF, RP, BP, GH, VT, RE, MH, and HT, as well as the overall quality of life in MCS might get benefit from taking G.H.3. tablets. The average levels of low moods and anxiety concerns were both relieved and the prevalence rate of clinical anxiety concern was reduced as well. No serious, adverse reactions from taking G.H.3. were found in this study. These results may assist physicians in determining its clinical applications.

### Ethics Statement

The study was approved by the School of Public Health, Shanghai Jiaotong University. All patients provided written informed consent.

## Abbreviations

RCT: Randomized controlled trial
G.H.3.: Gerovital H3
SDS: Self-Rating Depression Scale
SAS: Self-Rating Anxiety Scale
SF-36 scale: 36-item Short-Form Health Survey
DMAE: Dimethylaminoethanol
PABA: Para-aminobenzoic acid
PF: Hysical functioning
RP: Role-physical
BP: Bodily pain
GH: General health
VT: Vitality
SF: Social functioning
RE: Role-emotional
MH: Mental health
HT: Health transition
PCS: Physical composite score
MCS: Mental composite score
SPSS: Statistical Package for Social Sciences
